# DBMLoc: a Database of proteins with multiple subcellular localizations

**DOI:** 10.1186/1471-2105-9-127

**Published:** 2008-02-28

**Authors:** Song Zhang, Xuefeng Xia, Jincheng Shen, Yun Zhou, Zhirong Sun

**Affiliations:** 1 MOE Key Laboratory of Bioinformatics, State Key Laboratory of Biomembrane and Membrane Biotechnology, Department of Biological Sciences and Biotechnology, Tsinghua University, Beijing 100084, China

## Abstract

**Background:**

Subcellular localization information is one of the key features to protein function research. Locating to a specific subcellular compartment is essential for a protein to function efficiently. Proteins which have multiple localizations will provide more clues. This kind of proteins may take a high proportion, even more than 35%.

**Description:**

We have developed a database of proteins with multiple subcellular localizations, designated DBMLoc. The initial release contains 10470 multiple subcellular localization-annotated entries. Annotations are collected from primary protein databases, specific subcellular localization databases and literature texts. All the protein entries are cross-referenced to GO annotations and SwissProt. Protein-protein interactions are also annotated. They are classified into 12 large subcellular localization categories based on GO hierarchical architecture and original annotations. Download, search and sequence BLAST tools are also available on the website.

**Conclusion:**

DBMLoc is a protein database which collects proteins with more than one subcellular localization annotation. It is freely accessed at .

## Background

Knowledge of subcellular localization is crucial to understanding protein function and biological process. During translation or later, proteins will be transported into different compartments such as cytoplasm, membrane system, mitochondrion, etc., or may be secreted out of the cell. Locating to a specific subcellular compartment is essential for a protein to function efficiently. High-throughput experimental approaches like immuno-localization[1], tagged genes and reported fusions[2,3] have made the growth of localization data catch up with the avalanche of protein data. Swiss-Prot is a comprehensive database which includes subcellular localization information. In the recent years, some specific subcellular localization databases are constructed based on experimentation, computational prediction or both. The subcellular localization data of LOCATE[4] are from high-throughput immunofluorescence-based assay and publications. Organelle DB[5] annotates all protein localizations using vocabulary from the Gene Ontology consortium which facilitates data interoperability. DBSubLoc[6] uses a keyword-based system to integrate Swiss-Prot subcellular localization annotations. LOCtarget[7] and PA-GOSUB[8] implement predictors of subcellular localization based on different methods have been reported. PSORTdb[9] is a database for bacteria that contains both information determined through laboratory experimentation (ePSORTdb) and computational predictions (cPSORTdb). Eukaryotic database, eSLDB[10], collects five species' location data which are experimental-determined, homology-based or predicted. In addition, some bioinformatics methods have been developed to predict the protein subcellular location, which make use of the sorting signals[11], domain information[12], amino acid composition in the sequences [13-15] or other information[16].

However, a lot of proteins have more than one subcellular localization annotations. These proteins may simultaneously locate or move between different cellular compartments, for example, transcription factors and signaling pathway transduction factors. Proteins may play different roles in biological process when they are in different subcellular localizations. For these proteins, single subcellular localization annotation will lose some important information. Usually these proteins have more important biological functions. Their localization annotations will provide more valuable clues to researchers. These proteins are quite common, accounting for about 39% of all organellar proteins in mouse liver[17]. However, there are very few proteins annotated with multiple locations in the available subcellular localization databases. Here we have built the database DBMLoc which collects proteins with multiple subcellular localization annotations. It provides useful information for protein functional research as well as computational prediction. In addition, taxonomy, Swiss-Prot, GO and interaction information are also annotated. If protein has interactions, a subcellular localization quality score is computed on the basis of its interaction proteins' locations.

## Construction and content

The DBMLoc database is mainly developed from primary protein databases (Swiss-Prot/TrEMBL[18]), available experimental-determined subcellular localization databases (DBSubloc[6], ePSORTdb[9], MitoProteome[19], Organelle DB[5] and LOCATE[4]) and some literature references. Only full-length and unambiguous proteins are selected from Swiss-Prot, and those whose subcellular localization annotations are marked with "by similarity", "probable", "possible", "potential", "may be" are excluded. At the same time, multiple annotations are collected from subcellular localization databases (DBSubloc, ePSORTdb, MitoProteome, Organelle DB and LOCATE), then they are mapped to the protein set derived from Swiss-Prot. The redundant annotations are filtered. In order to standardize subcellular localization annotation terms, various terms of cellular compartments and complexes are assigned into twelve large organelle categories as follows: extracellular, cell wall, membrane, cytoplasm, mitochondrion, nucleus, ribosome, plastid, endoplasmic reticulum, Golgi apparatus, vacuole and virion. Cell wall, plastid and vacuole are unique in plant cell. Some subcellular localization annotations which can not be classified into the twelve categories are assigned into "others". There are 616 proteins that have "others" annotations. This process is mainly based on the Gene Ontology[20] annotations and original subcellular localization annotations. We annotate the proteins with GO ID from their primary sources or the annotation tools provided by GOA (Gene Ontology Annotation Database)[21]. The proteins are also cross-referenced to the NCBI Taxonomy database[22]. Sub-datasets are derived based on their taxonomy class (i.e. animal, plant, eukaryote, etc.)

Proteins that interact with each other tend to share the same subcellular localizations, so we annotate the protein with interaction data collected from DIP[23], MINT[24] and BIND[25]. To check the subcellular localization annotation quality, if it has interaction proteins, a quality score is computed based on the following formula. The higher the score is, the more reliable the subcellular localization annotations are. All the proteins whose score equals 1 are integrated into a high quality dataset.

$$Score=\frac{N1}{N2}$$

*N1*: Number of the localizations shared by its interaction proteins' subcellular localizations.

*N2*: Number of protein's subcellular localizations.

Finally, with some literature annotated proteins added, 10470 protein entries are integrated into DBMLoc database. The downloadable DBMLoc database and non-redundant sub-datasets are released as plain text files. The format is similar to that of Swiss-Prot data file. Each line in the file is one record of an entry in the 'KEY VALUE' format. The cross-reference records begin with a 'CX' key. Each of the value data contains one cross-reference record in the 'Reference Database: Reference ID' format, for example, the 'CX SWISS-PROT: Q85FL3' record means that the protein entry is linked to SWISS-PROT database Q85FL3 entry. More detailed description of the format can be found on the web page.

## Utility and discussion

**Figure 1:**
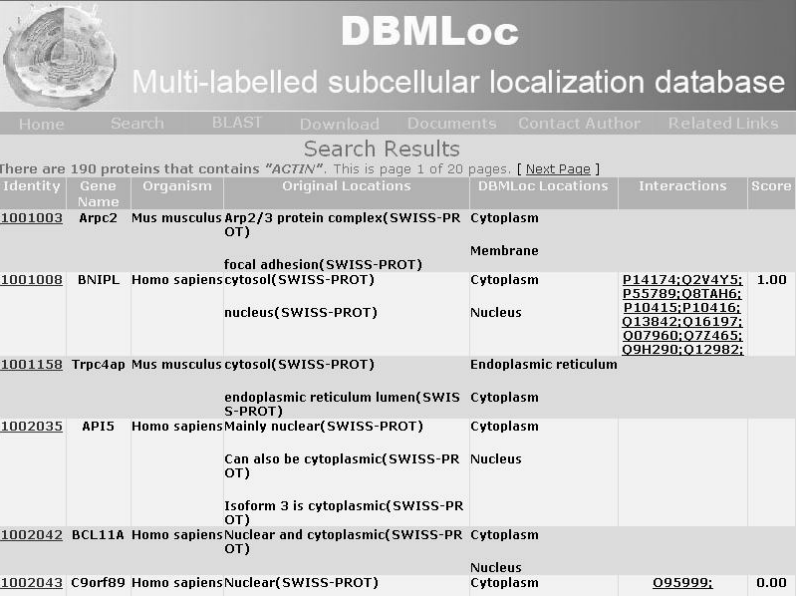
Protein name search result with keyword "actin".

**Figure 2:**
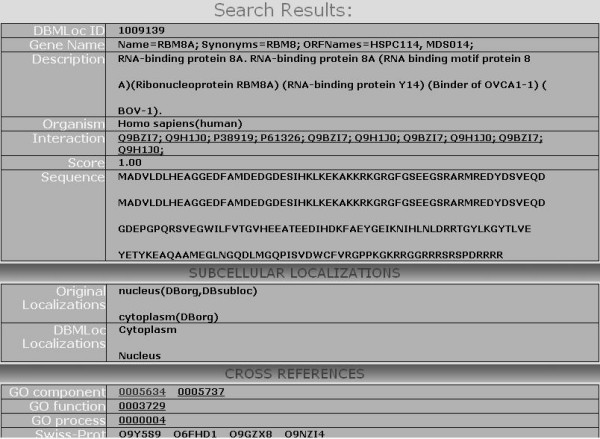
Swiss-Prot identity search result with query "Q9Y5S9".

We provide free download of the database, organism specific sub-datasets and taxonomy-categorized files for all the education and research users. Users can search the database with DBMLoc identity, cross-referenced database identity or protein name. Figures 1 and 2 show the name and identity search results. Protein sequence also can be submitted to search for homologous proteins in the full DBMLoc database or in one of its subsets.

The initial release contains 10470 multiple subcellular localization-annotated protein entries. Non-redundant protein data sets with sequence similarity less than 90% and 25% are also generated by BLAST. Table 1 lists brief statistical information on full and non-redundant data sets. The detailed statistical information is on the web page.

**Table 1:** Brief statistics of DBMLoc

	Full data sets	Non-redundant data sets (90%)	Non-redundant data sets (25%)
Two subcellular localizations	8887	6055	2366
Three subcellular localizations	1461	1112	593
Four subcellular localizations	107	100	85
Eukaryote	9954	6727	2549
Animal	6492	4240	1523
Plant	3462	2487	1278

Various databases' annotations integrated together in DBMLoc database might be false annotations or conflicts. So, we will pay more attention to the quality of data in the future development. More experimental data and other available information, like experimental method and post-translation modification, will be integrated to the database. The database will be updated regularly as new version of Swiss-Prot is available. Besides, more web services and analysis tools will be developed.

## Conclusion

DBMLoc is a specific database aimed at multiple localization annotated proteins. Proteins are cross-referenced to NCBI taxonomy, Gene Ontology and original database. Proteins that interact with each other tend to share the same subcellular localizations. So, protein-protein interaction information is also integrated into the database. A quality score is derived from protein-protein interactions. These data will be valuable to help experimental and computational biologists understand and analyze biological function.

## Availability and requirements

DBMLoc home page: 

License: The database is freely available.

## List of abbreviations

GO: Gene Ontology.

## Authors' contributions

SZ and XX designed and constructed the database. SZ drafted the manuscript. JS and YZ participated in data curation. ZS supervised the project. All authors read and approved the final manuscript.
